# Robotic-assisted simultaneous resection of a left-sided thymic neoplasm and a right-sided lower thoracic paravertebral neoplasm via the same ports and two docking steps: a case report

**DOI:** 10.1186/s13019-019-0989-5

**Published:** 2019-10-29

**Authors:** Hanlu Zhang, Zihao Wang, Yu Zheng, Fuqiang Wang, Yingcai Geng, Long-Qi Chen, Yun Wang

**Affiliations:** Department of Thoracic Surgery, West China Hospital of Sichuan University, Chengdu, 610041 China

**Keywords:** da Vinci Surgical System, Mediastinal neoplasms, Paravertebral neurogenic tumor

## Abstract

**Background:**

The authors presented a 63-year old female synchronously complicated with a thymic tumor located at the left-side of the superior mediastinum, and a paravertebral tumor located at the right-side of the lower thorax. Conventional thoracoscopic surgical procedure using rigid instruments to simultaneously resect the two tumors via the same ports might be technically challenging. To our knowledge, the use of a surgical robot allowed the surgeon to perform precise dissection from extreme angles with the characteristic of articulating surgical instruments.

**Case presentation:**

Two lesions were successfully dissected using the da Vinci Surgical System through the same four ports on the right side of the chest and two-step docking. Firstly, the patient cart came from the dorsal side of the patient and the paravertebral neoplasm was dissected. Afterwards, the patient cart was undocked and the operation table was rotated 180 degrees counterclockwise. The robot was re-introduced and the patient cart came from the ventral side of the patient and the whole thymus was resected.

**Conclusion:**

This case report suggests that two-step docking via the same four ports for these two tumors located at different directions of the thorax was safe and effective, demonstrating a clear advantage of the surgical robot.

## Background

Anterior mediastinal tumors usually arise from the thymus and paravertebral masses usually originate from the nerve sheath. Generally, these tumors are well encapsulated and typically treated using complete resection with excellent outcomes. Currently, resection of anterior mediastinal or paravertebral neoplasm is typically performed using minimally invasive procedures. Intriguingly, the present case suffered from a left-sided thymic tumor and a right-sided lower thoracic paravertebral neoplasm. Accordingly, usual minimally invasive surgical procedure with rigid instruments to simultaneously resect the two tumors via the same ports might be technically challenging. Given that the surgical robot can provide magnified three-dimensional images, articulating forceps and tremor filtering, which could potentially facilitate simultaneous excision of the thymic neoplasm and the paravertebral tumor via the same ports. The da Vinci robotic system was introduced for the resection.

## Case presentation

**Fig. 1:**
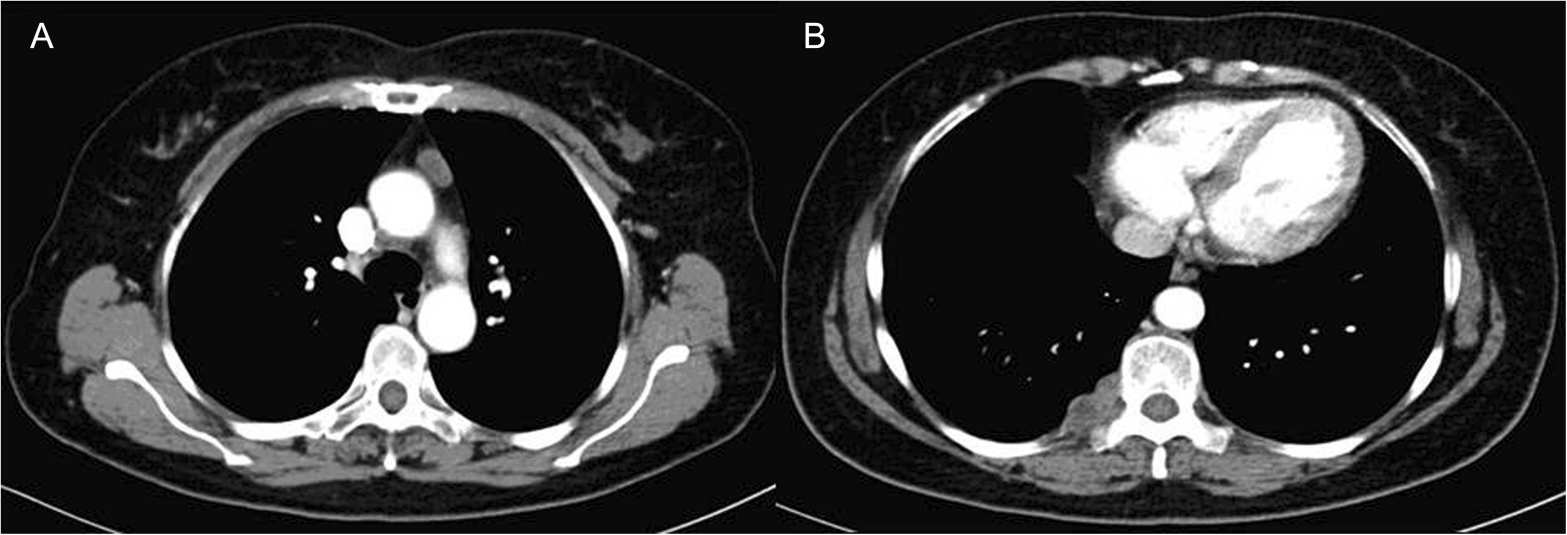
CT scan of the chest revealed that two tumors located at the left-sided anterior mediastinum (**a**) and paravertebral right-sided lower thorax (**b**), respectively

**Fig. 2:**
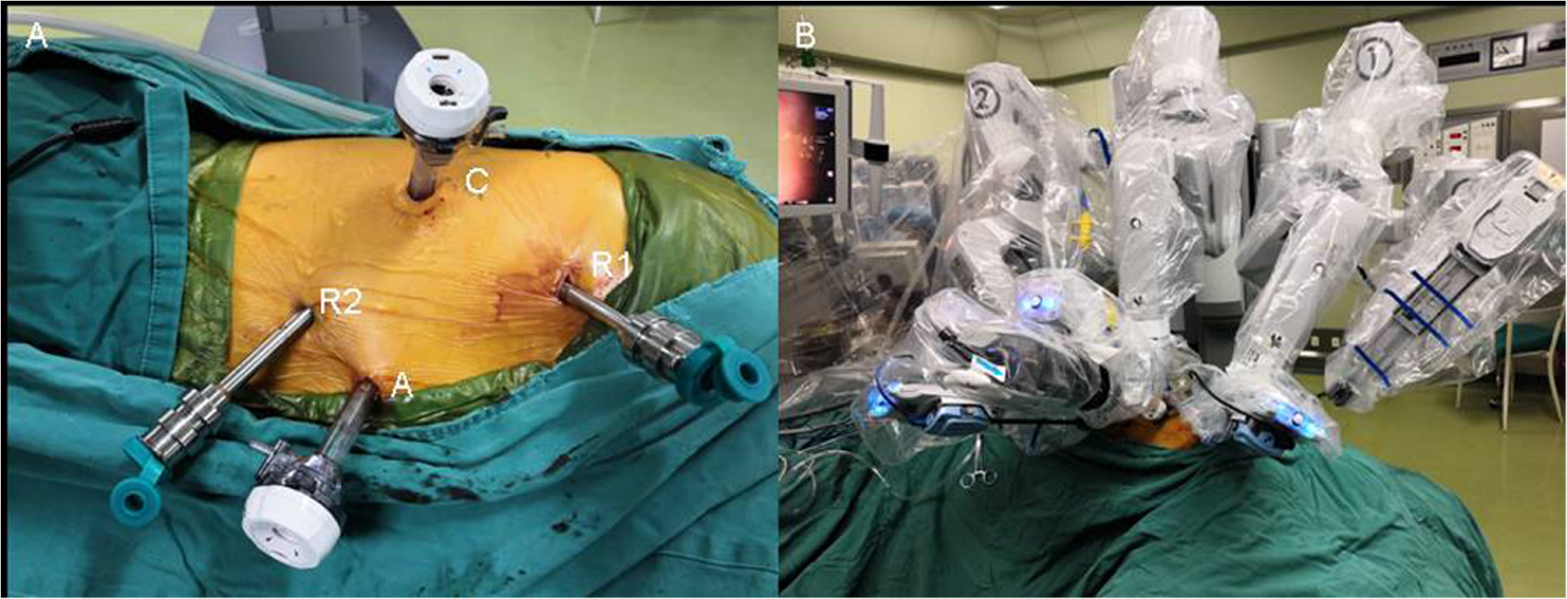
Port sites (**a**) and patient cart position (**b**) for the resection of the posterior mediastinal tumor. **c** port site of the camera, R1: port site of robotic arm 1, R2: port site of robotic arm 2, **a** port site for the assistant

**Fig. 3:**
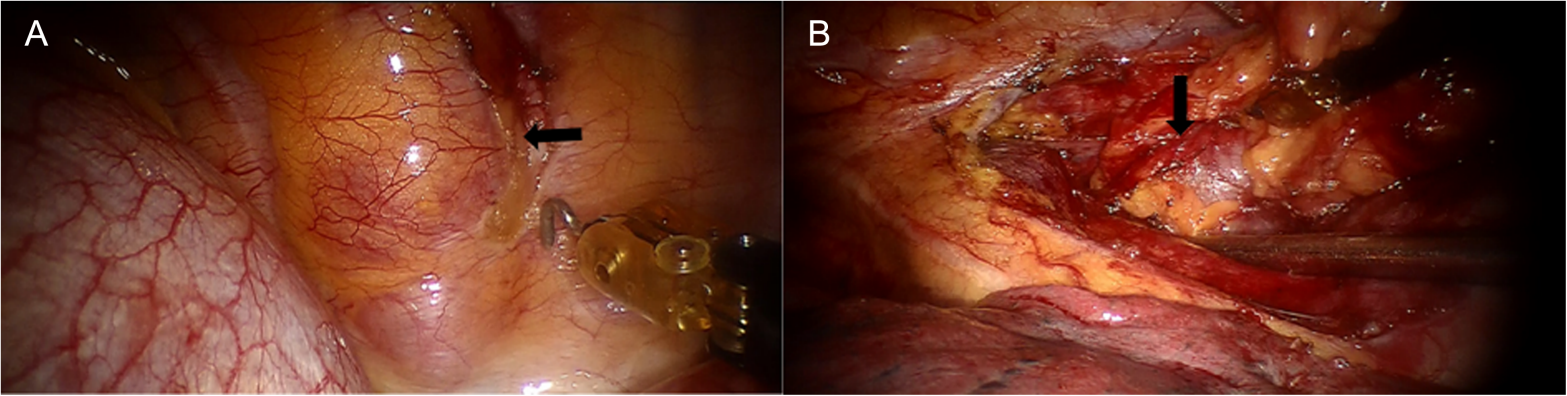
Right-sided paravertebral neoplasm (**a**) and tumor of the anterior mediastinum (**b**)

**Fig. 4:**
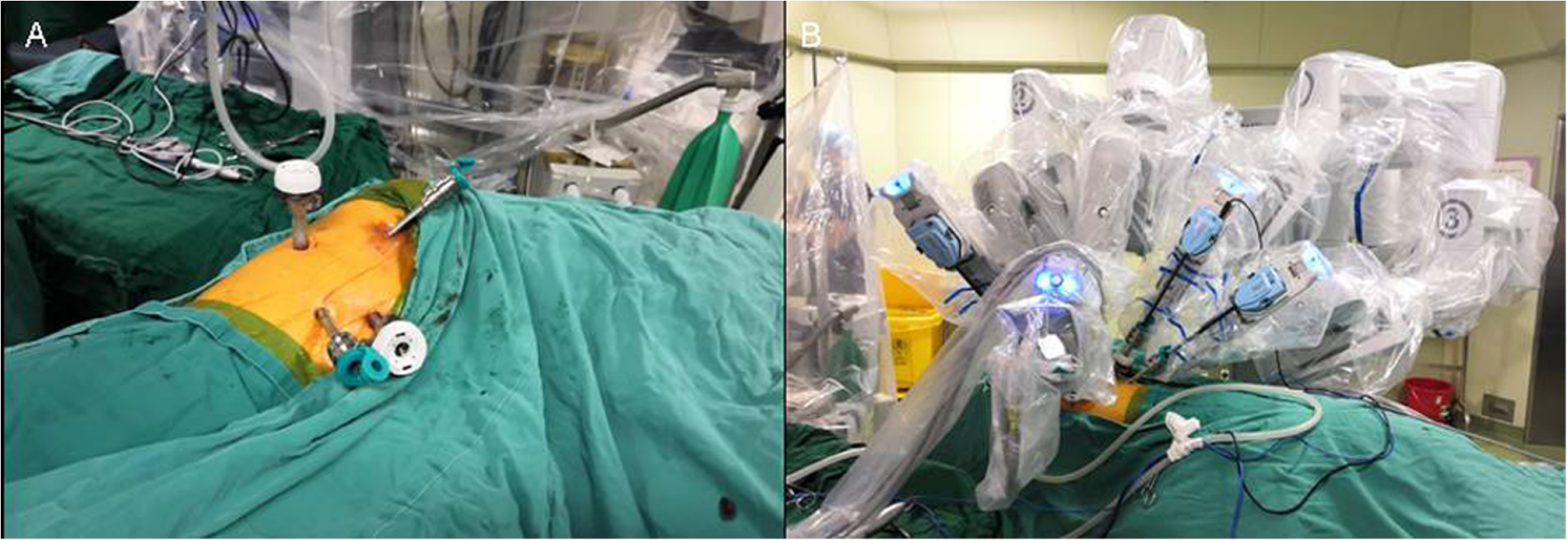
Port sites (**a**) and patient cart position (**b**) for the resection of the anterior mediastinal tumor. **c** port site of the camera, R1: port site of robotic arm 1, R2: port site of robotic arm 2, R3: port site for robotic arm 3

A 63-year-old female was referred to our outpatient department for routine checkup and chest computed tomography scans revealed two tumors measuring 18 × 9 mm and 33 × 15 mm in diameter, located at the left-sided anterior mediastinum (Fig. 1a) and paravertebral right-sided lower thorax (Fig. 1b), respectively. The initial diagnosis was a thymic tumor and a right-sided paravertebral neurogenic tumor. Taking into consideration the two tumors located at different directions of the thorax, it was determined that using a surgical robot via the right side of the thorax might be optimal to excise the two tumors. Under general anesthesia, with bilateral lung ventilations, the patient was placed in a left lateral decubitus position. Resection of the paravertebral tumor was performed at the first stage. In detail, a 12-mm port for camera was placed at the sixth intercostal space anterior to scapula angle. Two 8-mm trocars were inserted for the robotic instruments: one for the robotic arm 1 was inserted at the 3rd intercostal space anterior to scapula, and the other trocar for the robotic arm 2 was inserted at the 6th intercostal space in the right-side middle axillary line. A 12-mm trocar for the bedside assistant was placed at the 5th intercostal space in the right-side anterior axillary line (Fig. 2). Insufflation with CO2 was used at a pressure of 8 mmHg to deflate the right lung and the diaphragm. The right paravertebral tumor was located at the ninth vertebral body (Fig. 3a). The mass was dissected away from the surrounding structures with an intact capsule without laminectomy or facetectomy. Thereafter, the patient cart was undocked and the operation table was rotated 180 degrees counterclockwise. The patient position and port placements were identical to the previous configuration. The robotic cart was re-introduced. Resection of the whole thymus was performed during the second stage (Fig. 3b). Robotic arms 1, 2, 3 were then docked at the 5th intercostal space in the right-side middle axillary line, the 3rd intercostal space anterior to scapula, and the 6th intercostal space in the right-side anterior axillary line, respectively (Fig. 4). The trocar for arm 3 could be used for the table-side assistant if necessary. After completing the resection, the specimens were removed through the assistant port incision. Haemostasis was checked, followed by insertion of a chest tube.

**Fig. 5:**
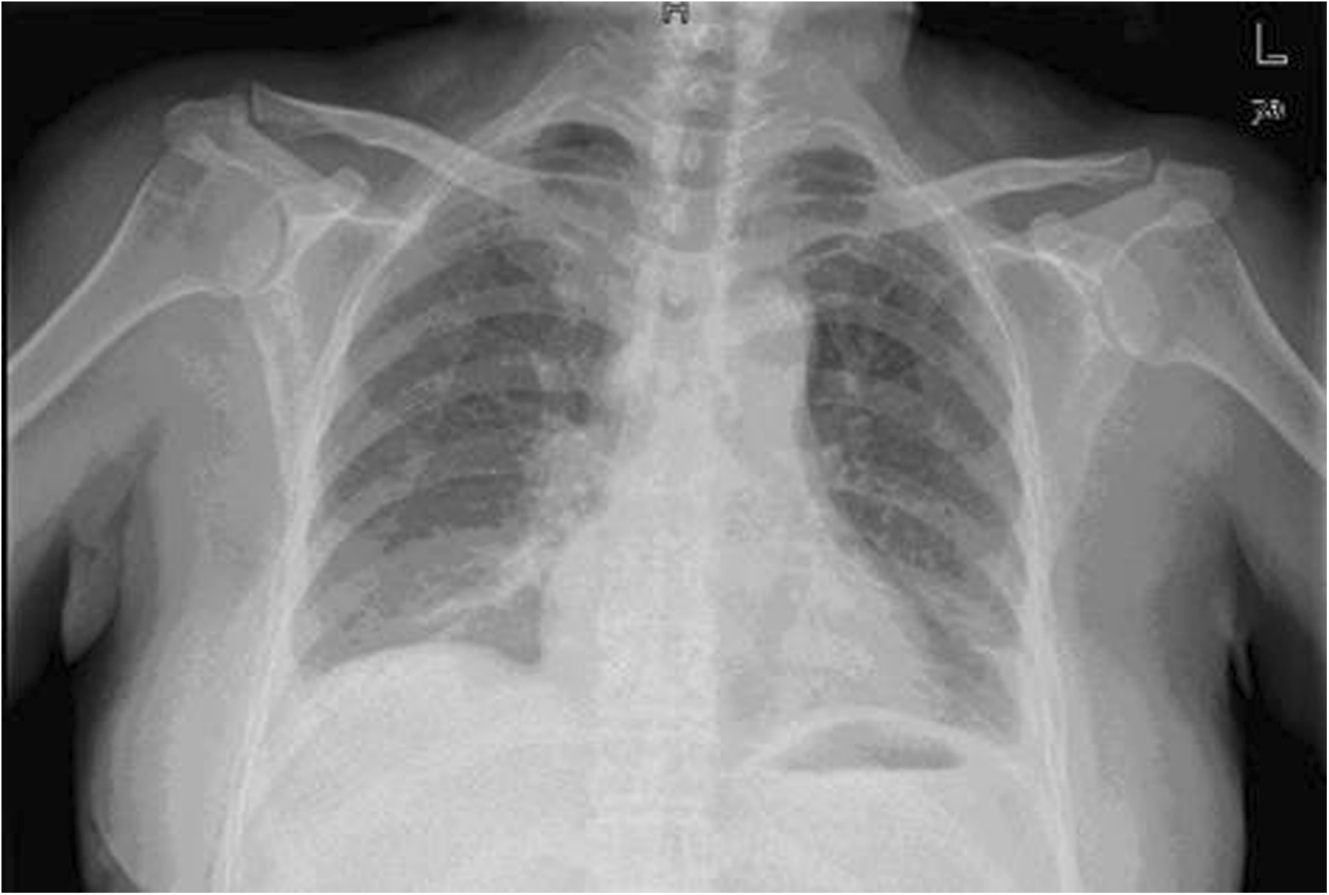
Postoperative chest radiograph

Total operating time was 180 min and the blood loss was less than 15 ml. The post-operative course was uneventful with no evidence of paralysis or motor weakness. The chest tube was removed on the following day, the chest radiograph was normal (Fig. 5) and the patient was discharged on the 3^rd^ post-operative day. Final pathological diagnoses of the anterior mediastinal tumor and paravertebral tumor were a bronchogenic cyst and a schwannoma, respectively, both of which had intact capsules.

## Discussion and conclusion

Although there are various potential technical advantages of robots over conventional thoracoscopy, Grimminger and colleagues [1] hold the view that surgical robot and thoracoscopy were both minimally invasive approaches that applied the same steps and approaches. The benefits of the robot are therefore not fully realized. However, the presented case demonstrated that da Vinci Surgical System might be a promising alternative to traditional thoracoscopic surgery in selected cases.

The primary treatment for paravertebral schwannoma and thymic bronchogenic cyst is surgical resection. Minimally invasive operations for mediastinal tumors have been widely accepted and recommended because of its clinical benefits in terms of lower blood loss, reduced length of stay and less postoperative pain compared to open surgery. However, the presented rare case was simultaneously complicated with a left-sided thymic neoplasm and a right-sided lower thoracic paravertebral neoplasm. To our knowledge, traditional thoracoscopic surgical procedures with rigid instruments to simultaneously resect the two tumors via the same ports might be challenging. However, the use of a surgical robot represents the most significant advancement in minimally invasive surgery and its application allows precise dissections from extreme angles in isolated areas that are difficult to access. Robotic surgery is regarded as an ideal procedure for mediastinal tumors with the advantages of having a stable operator-controlled camera, a high-definition 3D magnified view, articulating instruments with seven degrees of freedom and tremor filtration [2, 3].

Based on our experience, optimal port placement was important for simultaneous resection of the two tumors. We should not only avoid arm collisions, but also provide an optimal access for the robot camera and the instruments during the dissection process of the two tumors. Since major vascular (such as the left innominate vein or superior vena cava) injury is a catastrophe during robotic thoracoscopic thymectomy, port placement for the presented case was primarily based on the port arrangements for thymectomy. To provide an optimal visualization and instrument movement for the paravertebral tumor dissection, the usual port position for thymectomy was moved slightly backwards.

Robotic surgery is a time-consuming procedure due to the time required to dock, undock and exchange instruments. In our opinion, the duration of surgery can be shortened with the experience of more cases. Due to the high cost of installation and maintenance, cost-effectiveness is a practical burden associated with the introduction of the da Vinci Surgical System. Most patients could not afford to the cost of robotic surgery without health insurance coverage. Over the years, many companies have tried to develop competing robotic devices which may challenge the monopoly situation of da Vinci surgical robots. A cost reduction of robotic surgery is expected in the future.

## Data Availability

All the data and images is available for review by the Editor-in-Chief of this journal.

## References

[1] Grimminger PP, van der Horst S, Ruurda JP, van Det M, Morel P, van Hillegersberg R (2018). Surgical robotics for esophageal cancer. Ann N Y Acad Sci.

[2] Marulli G, Comacchio GM, Schiavon M, Rebusso A, Mammana M, Zampieri D (2018). Comparing robotic and trans-sternal thymectomy for early-stage thymoma: a propensity score-matching study. Eur J Cardio-thorac Surg.

[3] Pacchiarotti G, Wang MY, Kolcun JPG, Chang KH, Al Maaieh M, Reis VS (2017). Robotic paravertebral schwannoma resection at extreme locations of the thoracic cavity. Neurosurg Focus.

